# Clinical utility of high-flow nasal cannula oxygen therapy for acute respiratory failure in patients with hematological disease

**DOI:** 10.1186/s40064-016-2161-1

**Published:** 2016-04-23

**Authors:** Kaito Harada, Shuhei Kurosawa, Yutaro Hino, Keita Yamamoto, Masahiro Sakaguchi, Shuntaro Ikegawa, Keiichro Hattori, Aiko Igarashi, Kyoko Watakabe, Yasushi Senoo, Yuho Najima, Takeshi Hagino, Noriko Doki, Takeshi Kobayashi, Kazuhiko Kakihana, Toshihiro Iino, Hisashi Sakamaki, Kazuteru Ohashi

**Affiliations:** Hematology Division, Tokyo Metropolitan Cancer and Infectious Disease Center, Komagome Hospital, 3-18-22 Honkomagome, Bunkyo-ku, Tokyo, 113-8677 Japan; Medical Engineering Division, Tokyo Metropolitan Cancer and Infectious Disease Center, Komagome Hospital, Tokyo, Japan

**Keywords:** High-flow nasal cannula, Hematological disease, Acute respiratory failure

## Abstract

A high-flow nasal cannula (HFNC) is a newly developed device that enables high-flow oxygen therapy for patients with serious cardiopulmonary problems, but there are few data regarding its use in patients with hematological disease. The efficacy and tolerability of HFNCs for patients who developed ARF during the treatment of various hematological diseases was evaluated. Fifty-six patients underwent HFNC therapy during the last 2 years, and the causes of ARF were mainly pneumonia (n = 37) or acute congestive heart failure (n = 7). Only 11 patients (20 %) showed a good response to HFNC therapy, and remaining 45 patients (80 %) failed to respond to the initial HFNC therapy and, therefore, underwent second-line therapy including endotracheal intubation with mechanical ventilation (n = 15), non-invasive positive pressure ventilation (n = 1), or narcotic palliation alone (n = 29). Thus, HFNC appear not to be a viable treatment option in 4 out of 5 patients in this cohort of patients with hematological disease, but it was well tolerated in most patients (96 %); no major complications except for nasal soreness (n = 2) were observed. Multivariate analysis showed that the cause of ARF (pneumonia, odds ratio 11.2, 95 % CI 1.76–71.5, p = 0.01) was the only risk factor for treatment failure.

## Background

Patients with hematologic diseases often develop acute respiratory failure (ARF) as a result of intensified therapy or subsequent immunosuppression (Schuster and Marion 1983). Based on the current understanding, non-invasive positive pressure ventilation (NPPV) might provide more favorable outcomes, especially for immunocompromised patients, than conventional invasive ventilation (Azoulay et al. 2001; Antonelli et al. 1998; Depuydt et al. 2004; Soares et al. 2005; Hilbert et al. 2001). Recently, a high-flow nasal cannula (HFNC) has been introduced as a new, non-invasive device, which not only supplies a high concentration of oxygen, but also generates a low level of positive airway pressure (PEEP; positive end-expiratory pressure) (Roca et al. 2010; Ward 2013; Parke et al. 2011; Corley et al. 2011). Moreover, it can reduce ventilatory requirements by flushing the anatomical dead space with high-concentration oxygen, and well-humidified oxygen may facilitate secretion clearance (Roca et al. 2010; Ward 2013). Thus, this device would have many advantages over conventional oxygen therapy or NPPV, and, therefore, it has been assessed in various clinical settings (Chatila et al. 2004; Carratalá Perales et al. 2011; Messika et al. 2015; Sztrymf et al. 2011, 2012). However, there are few data about its use in hematological disorders, especially in a low platelet count setting (Gristina et al. 2011; Peters et al. 2013; Kang et al. 2015). A retrospective study to evaluate the efficacy, safety, and tolerability of HFNC therapy in patients with hematological diseases was performed at a single institution.

## Method and definitions

A retrospective chart review was performed to evaluate adult patients with various hematologic diseases who underwent HFNC therapy for treatment of ARF at our institution between October 2012 and September 2015. ARF was diagnosed when the patient met one of the following criteria under the condition of receiving oxygen supplementation at more than 4 L/min: oxygen saturation level <90 %; respiratory rate more than 25 breaths/min; and obvious signs of respiratory distress such as dyspnea, accessory-muscle use, and diaphoresis (Parke et al. 2011). Successful HFNC treatment was defined as when patients had been weaned from the HFNC without exacerbation of ARF. Treatment failure was simply defined as patients who failed to respond to HFNC therapy and underwent second-line therapy, including endotracheal intubation with mechanical ventilation or NPPV, or died on HFNC. HFNC therapy was judged as tolerable when patients had no HFNC-derived distress. The HFNC device (Optiflow™, MR850™ system, Fisher & Paykel Health-care, Auckland, New Zealand) consists of an air-oxygen blender, supplying an accurate fraction of delivery oxygen (FDO_2_) between 0.21 and 1.0, and a heated humidifier that allows the delivery of up to 60 L/min. This system should maintain the inspired gas at a temperature of 37 °C and an absolute humidity of 44 mg H_2_O/L (Roca et al. 2010). All patients were supplied with a median 10 L/min (range 4–20 L/min) of supplemental oxygen via nasal cannula or facial mask before putting on the HFNC. The initial HFNC settings were: median FDO_2_ 60 % (range 30–100 %); median HFNC flow 40 L/min (range 15–60 L/min); and median duration of HFNC therapy 88 h (range 1–950 h).

This study was approved by the institutional ethics committee of our institution.

### Statistical analysis

To evaluate the risk factors for HFNC treatment failure, the following categorical variables were used: age, sex, disease condition, the cause of ARF (pneumonia or not), neutropenia <500/µL, thrombocytopenia <30,000/µL, FDO_2_, concomitant clinical conditions including acute kidney injury, liver dysfunction, or allogeneic hematopoietic stem cell transplantation (allo-HSCT), past clinical history including allo-HSCT, heart diseases, or pulmonary diseases, and the amount of delivered oxygen via nasal cannula or facial mask before starting HFNC therapy. Variables with p < 0.10 were entered into the full model. On univariate analyses, Fisher’s exact tests (categorical variables) or Mann–Whitney U tests (continuous variables) were carried out. Multivariate logistic regression analysis was performed by a forced entry, and both odds ratios and associated 95 % confidence intervals (95 % CI) were calculated. In comparison of vital signs before and after initiating HFNC, Wilcoxon signed-rank test was carried out in terms of heart rate, oxygen saturation, and respiratory rate. All p values were two-sided. p values <0.05 were considered significant.

## Results

During the last 3 years, 56 patients eventually underwent HFNC treatment for ARF in our institution. The median age was 59 years (range 24–82 years), and 38 patients were men (68 %). The underlying diseases were mostly hematologic malignancies and other clinical characteristics are summarized in Table 1.

**Table 1:** Characteristics of patients who underwent HFNC therapy

Number (n)	56
Median age, years (range)	59 (24–82)
Sex (male/female)	38/18
Underlying hematological disease (n)	
AML	23
ALL	9
MDS	11
CMML	2
CMLBC	2
ATLL	3
NHL	3
PLL	1
ITP	1
SAA	1
Disease risk^a^ (high/low)	33/23
Cause of ARF (n)	
Pneumonia	37
Congestive heart failure	7
Organized pneumonia	4
Pulmonary chronic GVHD	2
Leukemic pulmonary invasion	2
Multiple organ failure	2
No identifiable cause	2
Median number of WBC (/μL) at the onset of ARF (range)	1850 (10–398,300)
Neutropenia (<500/μL)at the onset of ARF (yes/no)	32/24
Median number of platelet at the onset of ARF (×10^4^/μL) (range)	2.8 (0.2–28.8)
Concomitant clinical condition	
Acute kidney injury^b^ (yes/no)	35/21
Liver dysfunction^c^ (yes/no)	14/42
Allogeneic hematopoietic stem cell transplantation (yes/no)	26/30
Past clinical history	
Cardiac disease^d^ (yes/no)	5/51
Pulmonary disease^e^ (yes/no)	8/48
Allogeneic hematopoietic stem cell transplantation (yes/no)	42/14
Median oxygen supplement volume before putting HFNC (L/min) (range)	10 (4–20)
HFNC setting	
Median FDO_2_ (%) (range)	60 (30–100)
Median flow (L/min) (range)	40 (15–60)
Median time used (h) (range)	88 (1–950)

*HFNC* high-flow nasal cannula, *AML* acute myeloid leukemia, *ALL* acute lymphoid leukemia, *MDS* myelodysplastic syndrome, *CMML* chronic myelomonocytic leukemia, *CMLBC* chronic myeloid leukemia blast crisis, *ATLL* adult T cell leukemia and lymphoma, *NHL* non-Hodgkin’s lymphoma, *PLL* prolymphocytic leukemia, *ITP* idiopathic thrombocytopenic purpura, *SAA* severe aplastic anemia, *ARF* acute respiratory failure, *GVHD* graft-versus-host disease, *WBC* white blood cell, *FDO*_*2*_ fraction of delivery O_2_

^a^Disease risk was classified into two categories; high risk included acute leukemia not in remission, myelodysplastic syndrome with excess blast count, chronic myelomonocytic leukemia, or chronic myeloid leukemia blast crisis, the others were classified as low-risk

^b^Acute kidney injury was defined as having serum creatinine ≥1.5× upper limit of normal (ULN), or more than 0.3 points higher than baseline

^c^Liver dysfunction was defined as having serum aspartate aminotransferase/alanine aminotransferase ≥3× ULN, or total bilirubin ≥1.5× ULN (CTCAE ver. 4: grade 2)

^d^Past cardiac disease included atrial fibrillation, chronic congestive heart failure

^e^Past pulmonary disease included chronic obstructive pulmonary disease, bronchiolitis obliterans, forced expiratory volume% in 1 s ≤80 %, or diffusing capacity for carbon monoxide ≤80 %

### Outcomes of HFNC

Eleven patients (20 %) responded well to HFNC therapy and were successfully weaned from the HFNC without exacerbation of ARF, while 45 patients (80 %) failed to respond to initial HFNC therapy; they therefore underwent second-line therapy including endotracheal intubation with mechanical ventilation (n = 15), NPPV (n = 1), or narcotic palliation (n = 29). One patient with NPPV subsequently underwent endotracheal intubation with mechanical ventilation because of progressive hypoxia; therefore, 16 patients (29 %) eventually underwent endotracheal intubation with mechanical ventilation. However, only four of the patients undergoing mechanical ventilation survived. Thus, a total of 15 patients (27 %), 11 patients with HFNC and four patients with endotracheal intubation with mechanical ventilation, survived ARF.

Results of univariate and multivariate analyses of the risk factors for treatment failure are shown in Table 2. On univariate analyses, neutropenia and low platelet count (<3.0 × 10^4^/μL) at the onset of ARF, cause of ARF (pneumonia), and concomitant clinical condition of allo-HSCT were significantly related to the risk of treatment failure, whereas age, sex, disease risk, concomitant disease or condition except for allo-HSCT, past clinical disease or condition, and oxygen supplement volume before starting HFNC therapy (>10 L/min), FDO_2_ were not significant. Multivariate analysis with logistic regression identified the cause of ARF (pneumonia, odds ratio 11.2, 95 % CI 1.76–71.5, p = 0.01) as the only risk factor for treatment failure (Table 2).

**Table 2:** Risk factors^a^ for HFNC treatment failure

	Univariate analysis	Multivariate analysis		
	p value	p value	OR	95 % CI
Age (≥60 years)	1			
Sex (male)	0.31			
Neutropenia (yes)	0.02	0.81	1.40	0.09–20.8
Thrombocytopenia (<3.0 × 10^4^/μL) (yes)	<0.01	0.15	7.07	0.49–102
Disease risk (high^b^)	0.50			
Cause of ARF (pneumonia)	<0.01	0.01	11.2	1.76–71.5
Concomitant AKI (yes)	0.73			
Concomitant liver dysfunction (yes)	0.71			
Under allo-HSCT (yes)	0.05	0.52	2.13	0.21–21.7
Past history of cardiac disease (yes)	1			
Past history of pulmonary disease (yes)	1			
Past history of allo-HSCT (yes)	0.12			
FDO_2_ (≥60 %)	0.26			
Oxygen supplement volume before starting HFNC (≥10 L/min)	0.33			

*95* *% CI* 95 % confidence interval, *HFNC* high-flow nasal cannula, *ARF* acute respiratory failure, *AKI* acute kidney injury, *allo*-*HSCT* allogeneic hematopoietic stem cell transplantation, *FDO*_*2*_ fraction of delivery O_2_

^a^All statistical analyses were performed with EZR statistical software

^b^Disease risk was classified into two categories; high risk included acute leukemia not in remission, myelodysplastic syndrome with excess blast count or chronic myelomonocytic leukemia, the others were classified as low-risk

**Fig. 1:**
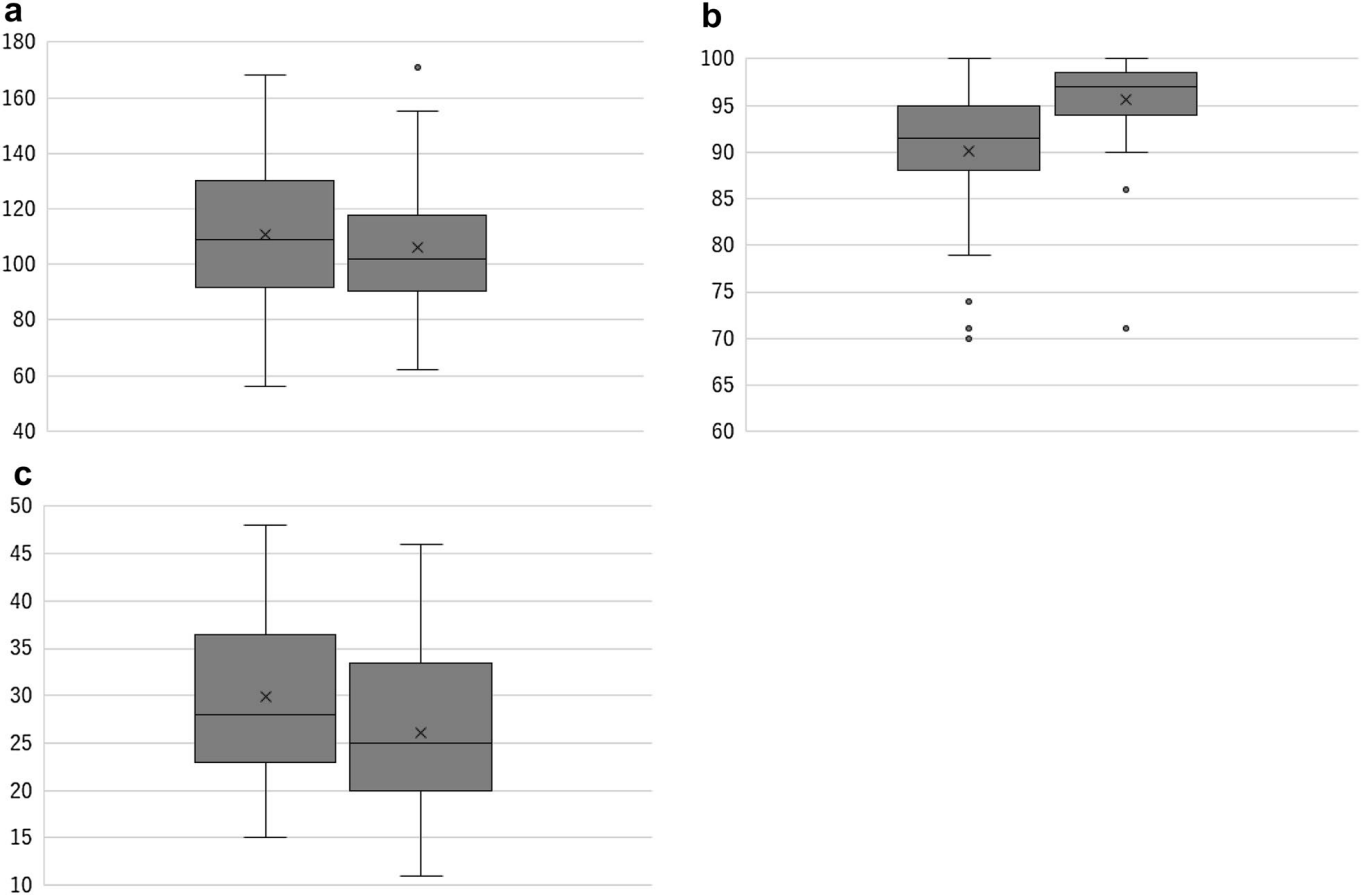
Comparison of vital signs before and after initiating high-flow nasal cannula (HFNC). Heart rate (**a**), oxygen saturation level (**b**), and respiratory rate (**c**) were significantly improved after initiating HFNC (p < 0.01 in all parameters)

Comparison of vital signs before and after initiating HFNC was shown in Fig. 1. Median heart rate (n = 44), oxygen saturation level (n = 50), and respiratory rate (n = 27) of patients with ARF before initiating HFNC were 109/min, 91.5 %, 28/min, respectively. These parameters were significantly improved after initiating HFNC. Median heart rate, oxygen saturation level, respiratory rate were 102/min (p < 0.01), 97 % (p < 0.01), 25/min (p < 0.01), respectively.

## Discussion

In the present cohort including a small number of patients evaluated retrospectively, HFNC therapy was well tolerated even in patients under or after allo-HSCT who were profoundly immunosuppressed or had low platelet counts and it significantly improved clinical vital signs of heart rate, oxygen saturation, and respiratory rate; however, the success rate was only 20 %, and 45 patients (80 %) subsequently underwent second-line therapy. The present success rate was low compared to previous reports in which it was up to 90 % (Messika et al. 2015; Peters et al. 2013; Parke et al. 2011; Frat et al. 2015). We assume that the discrepancy is likely attributable to different patient background characteristics. The present patients were severely ill, with a high simplified acute physiology score (SAPS) II (median 43, range 14–88) (Antonelli et al. 1998; Hilbert et al. 2000, 2001; Gristina et al. 2011). Moreover, most patients had concomitant medical conditions, including acute kidney injury (n = 35) or liver dysfunction (n = 14), or were under allo-HSCT (n = 26), as shown in Table 1. In addition, since patients who underwent palliation or died on HFNC were classified as a non-responder to HNFC regardless to the response to HFNC, we might underestimate the efficacy of HFNC.

Several reports have shown that invasive ventilation might be a risk factor for poor outcomes, especially in critically ill cancer patients; such patients are currently more likely to undergo non-invasive ventilation as initial treatment for ARF. However, few studies have directly compared the therapeutic efficacy of HFNC and NPPV. Kugelman et al. (2015) reported comparable data for the subsequent intubation rate between HFNC and NPPV therapy in neonatal patients. Frat et al. (2015) compared the intubation rate with conventional oxygen therapy, HFNC, or NPPV in a study. There were no differences in the intubation rate among the three groups. Focusing on selected patients who could be treated with PaO_2_/FiO_2_ ratios of < 200, a significant difference in the subsequent intubation rate between HFNC and other modalities was observed. In addition, the ICU mortality rate at 28 days was significantly lower with HFNC therapy (Frat et al. 2015).

Although two patients gave up HFNC therapy due to either agitation or restlessness, the HFNC was well tolerated in the remaining patients (96 %), and no major adverse events, such as nasal mucosal hemorrhage or necrosis, were observed, even though most patients had a low platelet count (median 3.5 × 10^4^/μL). As a minor adverse event, two patients complained of nasal soreness, but it was greatly relieved with a dose adjustment of the flow volume of oxygen.

With respect to the etiology of ARF, pneumonia (n = 37) was a major cause of ARF, and only seven patients with CHF underwent HFNC in the present series. Indeed, 12 of the present patients with pneumonia (32 %) developed ARDS, and only 2 of 37 patients (5 %) with pneumonia had successful treatment, while 9 of 19 patients (47 %) with other etiology recovered from ARF. Moreover, the present multivariate analysis clearly showed that pneumonia was a risk factor for treatment failure. Although Frat et al. (2015) showed more favorable outcome of HFNC treatment in pneumonia patients, their study excluded patients with neutropenia. The percentage of pneumonia related to immunosuppression was as low as 6 %. Thus, the discrepancy is likely attributable to different characteristics of patient and disease profile. Although further clarification is needed regarding which patients with ARF should undergo HFNC therapy and which conditions of HFNC therapy to use, pneumonia complicated with ARDS, which usually requires high pressure-PEEP for better management, might not be appropriate for HFNC therapy (Briel et al. 2010).

The present study had several limitations. The small size of the cohorts is one, and the retrospective nature of the study prevented the obtaining of missing data, including the arterial blood gas level before and after HFNC, which would be important parameters for risk analysis or clinical judgement. Nevertheless, the focus was to outline the clinical utility of HFNC therapy for ARF in patients with hematological disease. Although, further clarification is needed to determine whether HNFC therapy is better for patients with hematological disease with profound immunosuppression or severe thrombocytopenia, our data should provide useful insights into this new device.

## Conclusion

HFNC therapy was safe and well tolerated in patients with hematologic diseases who developed ARF, however, it appeared not to be a viable treatment option in 4 out of 5 patients and pneumonia could be the risk factor of treatment failure of HFNC.
